# On Medical Reform

**Published:** 1810-01

**Authors:** 


### ON MEDICAL REFORM.

AS the word *Reform* implies some defect or degeneracy from original perfection, or else a degree of imperfection incompatible with the present state of human knowledge, it is regarded in the light of an accusation. Such an indication of hostility, or as it is considered, declaration of war, rouses suspicion, examination, and a dread of its tendency, in many who may become the objects of it. Abuse, negligence, incapacity, avarice, all take the alarm; and the outworks of opposition are inspected and put into a state of defence. This is perfectly natural, what always has and will happen. The artillery commonly directed against all reform, is either the sacredness of the subject, the venerable antiquity of the present order of things, the danger of all innovation; and very often we are told that the fabric is too ruinous to admit of repair. Miserable, indeed, would be the state of human affairs, if these obstacles were generally allowed to impede reform or improvement, which is nearly synonimous with it. We know that *religion*, the most sacred of all subjects, has been repeatedly reformed; and *the Reformation*, as it is emphatically called, is the boast of one-third of Europe and America. I might easily show that the constitution of states, the administration of justice, the discipline of navies and armies, with many other subjects of less importance, have often undergone salutary reform; and then proceed to combat the too common apathy about, or frequent opposition against it; but I have, perhaps, digressed too far already. My object is to submit a few observations on the subject of reform, respecting the evidence of the due qualification of persons allowed to practise upon the *health* of his Majesty's subjects.

A Correspondent, in the last number of your Journal, who signs himself "a *licensed physician*," produces very weighty authorities to prove, that the Royal College of Physicians of London has a competent jurisdiction over, or at least a power of licencing, all persons who assume the liberty of adding M. D. to their names. Granting this to be true in *theory*, which I am by no means disposed to dispute, we will consider hereafter, whether any *practical* good results, has resulted, or is ever likely to result, from this authority of the College. But, in this place, I wish to call the attention of your readers and correspondents to the *other* branches of the profession; and to ask, whether whether the surgeon-apothecaries, and the chirurgo-obstetro-pharmacopolists, do not also require that some proof of ability and competence should be given, before they are allowed to practise in those respective branches?

I take it for granted that all your readers will confess, that the class of practitioners who arrange themselves under those denominations, are ten times, at least, as numerous as the *Doctors*; and, of course, the guarding his Majesty's subjects against their errors or ignorance, is of ten times greater importance than the regulation of the conduct of those called physicians. This conclusion does by no means rest on the mere *numbers* of that class of medical men, but much more on the circumstances under which they are called upon to decide. Few families, in the first instance, think it necessary to call in a physician; the family apothecary is deemed, and often with good reason, abundantly sufficient for a first decision. This first decision, however, is often of far more importance than all that can afterwards be suggested. The rash adoption, or the ignorant delay, of venesection, is well known to be very frequently fatal in acute diseases. But the most fatal effect of ignorance, arises from mistaking the nature and causes of a disease in the first instance; and the *difficulty*, in a majority of cases, of forming a correct decision in a first visit, is confessed by all writers and practitioners. How necessary, therefore, must it appear, that the persons on whom this diagnosis depends, should be duly qualified to form it? It has always appeared to me, that the most important part of medical reform should be directed to the lowest class of the Faculty; for I can hardly suppose that any person, who has obtained a diploma from a regular university, can be grossly ignorant of the distinctions of diseases. The present advocates for reform, appear to direct their chief attention to these inferior orders. The statutes, therefore, quoted by your correspondent, do only meet a small part of the question; and, consequently, cannot supersede the necessity of the proposed application to parliament.

Let us next consider whether the law, as it now stands, according to the statement of your correspondent, is of any practical utility in securing the due qualification, even of physicians, to whom alone it is applicable. It has hitherto remained a dead letter, because it contains no provision for enforcing its operation. If we had statutes which contained every desirable regulation for all the branches of the practice of medicine, but no practical

 method method of applying them, of what use would they be to the public? At present, it seems that any person may indite or inform against any one who practises as a physician, without having been examined and authorised by the president, and certain elects, of the Royal College of London. But who has hitherto been found willing to undertake so ungrateful an office? The name of informer, even when it is to bring acknowledged guilt to punishment, is so odious, that large rewards are hardly sufficient to procure them. In this case, the informer would subject himself to the imputation of envy or malevolence, without a motive to induce him; and hence it is, that none can be found to undertake the task.

This statute then of Henry the Eighth, quoted by your correspondent, is of no practical utility; and if it were, it applies only to a part of the profession, and the part least likely to injure the public through ignorance. For these reasons, therefore, I should approve of the intended application to parliament, if it were only for the means of enforcing the operation of the existing laws; but the object is far more extensive, as the bill will include every part of the profession, and will provide the means of securing its own operation. I can see no reason to conclude, with your correspondent, that by the passing of a comprehensive and operative bill, that "a very salutary existing law would be virtually abolished, and the public left at the mercy of any possessor of a purchased piece of parchment, called a diploma, without the possibility of redress." The new bill, on the contrary, instead of *repealing* any salutary existing law, will rouse it into activity from that sleep in which it has so long lain. I conclude, therefore, that instead of calling forth any "very formidable opposition to the bill from certain powerful public bodies," that those bodies will find "their ancient privileges maintained" and invigorated by it. I should, indeed, have very little expectation of any bill passing both Houses, that attempted to abolish the privileges of public bodies, which "they can *demonstrate to be of public utility.*"

That opposition, however, is to be expected, I cannot doubt; but from a very different quarter. The jealousy of some, the ignorance of others, and perhaps the avarice of a few, may excite opposition; but probably this will operate secretly, by applications and misrepresentations to individual members of parliament. If the bill be properly drawn, I cannot fear any formidable opposition from respectable public bodies.

If

If such a bill as that above supposed (for I am not a Member of the Lincolnshire Society), should be entertained, and sent to a Committee of the House of Commons, I believe it will be productive of good effects; because such instances of gross ignorance and incapacity will there be produced, as cannot fail to awaken public attention to the importance of the subject. A principle will be recognised, tending to show the great difference between trafficking in health and life, and the other articles of daily consumption. And if the whole object be not immediately attained, an important progress will, at least, be made towards it.

I am, &c.

AN EXTRA LICENTIATE.

*Dec.* 11, 1809.

